# Self-Amplifying RNA Vaccine Candidates: Alternative Platforms for mRNA Vaccine Development

**DOI:** 10.3390/pathogens12010138

**Published:** 2023-01-13

**Authors:** Christin Schmidt, Barbara S. Schnierle

**Affiliations:** Section AIDS and Newly Emerging Pathogens, Department of Virology, Paul-Ehrlich-Institut, 63225 Langen, Germany

**Keywords:** vaccine, mRNA, self-amplifying RNA, alphavirus, innate immunity

## Abstract

The present use of mRNA vaccines against COVID-19 has shown for the first time the potential of mRNA vaccines for infectious diseases. Here we will summarize the current knowledge about improved mRNA vaccines, i.e., the self-amplifying mRNA (saRNA) vaccines. This approach may enhance antigen expression by amplification of the antigen-encoding RNA. RNA design, RNA delivery, and the innate immune responses induced by RNA will be reviewed.

## 1. RNA Vaccines

Traditional vaccines are mainly based on inactivated or attenuated pathogens and need long development times, which makes a fast response to newly emerging pathogens difficult. However, vaccines based on nucleic acids can circumvent this shortcoming, and RNA was first suggested as a vaccine candidate more than 30 years ago [1]. The use of mRNA vaccines during the current COVID-19 pandemic has demonstrated their feasibility and clear advantages in combating emerging infectious diseases [2].

The production of mRNA vaccines is technically simple, fast, cost-effective, cell- and animal-material free, and easy to adapt [3] mRNA vaccines can be produced from linearized plasmid DNA templates or from polymerase chain reaction-based templates by in vitro transcription using phage RNA polymerases such as T7, T3, or SP6 polymerase [4] Cap structures can be added during in vitro transcription or enzymatically post-transcription [5,6]. All components are animal-material free; however, manufacturing has to be performed with RNase-free materials. Residual DNA is removed by DNase treatment and the resulting mRNA is further purified by bead-based methods, chromatography, or precipitation. These cell-free production steps can be easily standardized and upscaled to obtain clinical-grade material [7]. The production of RNA vaccine candidates is fast and an influenza vaccine candidate was reported to have been produced in only 8 days [8]. 

Moreover, in contrast to DNA-based vaccines, there is no risk of mRNA integrating into the host genome [9]. mRNA is non-infectious and only transiently present in cells due to its degradation by host cell RNases [10]. These safety properties have also been demonstrated by the COVID-19 mRNA vaccines, which show mainly only mild adverse events [11,12,13]. In contrast to vector vaccines, there is no preexisting anti-vector immunity, and immunizations with mRNA can be performed repeatedly [9]. Finally, COVID-19 mRNA vaccines have been shown to be efficacious and induce potent humoral and cellular immune responses [14]. 

**Figure 1:**
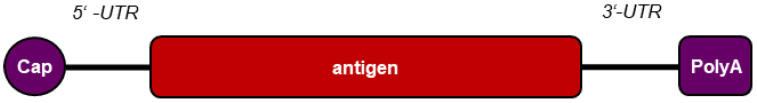
The structural elements of mRNA vaccines.

The mRNA vaccines consist of an open reading frame (ORF) encoding for the immunogenic antigen, a 5′- and a 3′-untranslated region (UTR), a 5′-cap structure, and a 3′-polyadenylated tail (poly-A) (Figure 1). Like cellular mRNAs, mRNA vaccines are directly translated in situ into the respective antigen. The transfer of mRNA to antigen-presenting cells allows the processing of the expressed antigen by the host cell proteasome and consequent loading onto major histocompatibility complex (MHC) class I molecules. Additionally, after ingestion of secreted antigens and degradation in the endosome, peptides can be presented by MHC class II molecules. Thereby, both CD8+ and CD4+ T cells can be stimulated, respectively. T cell activation further requires co-stimulatory molecules and cytokine secretion, which may result from innate immune sensing of mRNA vaccines (see below). Besides cellular immune responses, the humoral immune response can also be stimulated by the activation of B cells through secreted antigens. Consequently, antigen-specific humoral and cellular immune responses are induced by mRNA vaccination. 

To be efficacious, mRNA vaccines needed several alterations to enhance mRNA stability, modulate the innate immune responses to RNA, and enable efficient delivery of mRNA in vivo. Several elements were optimized: 

### 1.1. Increasing mRNA Stability and Translation Efficiency

The 5′- and 3′-UTR sequences from viral or eukaryotic genes modulate the half-life and translation of mRNAs, and therefore the UTR sequences were optimized for use in mRNA vaccination [15,16,17,18]. 

The 5′-UTR is primarily involved in recognition of the RNA by ribosomes and the translation of the downstream gene. A Kozak sequence is usually added to improve translation efficiency [19]. The 3′-UTR mainly regulates mRNA stability [20]. For example, the 3′-UTR of the highly expressed β-globin gene increases mRNA stability [15]. Novel 3′-UTR elements can also be identified by cellular library screenings [21].

Moreover, the length of the 3′-poly-A tail regulates mRNA stability and enhances translation [22]. The poly-A tail usually consists of around 200 units [23], but in dendritic cells (DCs) the average size is 120–150 nucleotides [24,25]. In addition, the poly-A tail interacts with the cap structure via the poly-A binding proteins and translation initiation factors, forming a loop. Highly expressed genes have been found to have short poly-A sequences and form loops efficiently [26]. The influence of poly-A tails on eukaryotic gene expression has been reviewed in [27]; however, the precise role of poly-A length in RNA vaccines still needs further investigation. In mRNA vaccines a poly-A tail of 100 nucleotides has been shown to be sufficient for efficient antigen expression and induction of immune responses [28,29].

One mechanism by which eukaryotic cells can differentiate between self and non-self mRNA is the cap structure [30]. The eukaryotic cap is a 7-methylguanosine (m7G) cap linked to the mRNA by a 5′-5′-triphosphate bridge (ppp) (m7GpppN structure) [31]. Cap-0 structures contain only the m7G cap. Further methylation of the 2′-hydroxy-groups of the first ribose moiety generates the cap-1 and additional methylation of the second ribose produces the cap-2 structure. Viral RNAs often contain cap-0 structures, and RNA containing cap-0 structures is recognized by the innate immune system (see below); therefore, cap-1/2 structures are superior for vaccine development. For example, in-vitro-transcribed mRNA with anti-reverse cap analogues (ARCAs; m27, 3′-OGpppG) have demonstrated enhanced translation [32,33]. Cap-1 structures can be added enzymatically by 2′-O-methyltransferase or co-transcriptionally by the CleanCap technology.

In addition, the antigen sequence can be codon optimized to increase antigen expression. For this purpose, synonymous mutations are introduced into the genes with the intention of eliminating rare codons that may slow translation efficiency. Codon optimization also affects GC content and RNA secondary structure, which both influence translation efficiency and can thereby be optimized [34].

### 1.2. In Vivo Delivery of mRNA Vaccine Candidates

For in vivo applications, RNA-based vaccines need to be taken up by cells. This passage through the phospholipid bilayer of the cell membrane is challenging due to their large molecular weight, negative charge, and fast degradation by nucleases. Different methods can be used for the in vivo delivery of mRNA.

The simplest delivery method is naked mRNA. It can be taken up by cells via scavenger receptor-mediated endocytosis; however, only small mRNA amounts are released into the cytoplasm [35]. Therefore, the uptake of mRNA is inefficient in most cell types, with the exception of immature DCs, which take up mRNA by micropinocytosis as part of their normal biological function. After intra-dermal or intra-nodal vaccination with naked mRNA, the mRNA is taken up by lymph node DCs, and inhibiting micropinocytosis abrogates internalization [36].

However, improved methods for delivery are needed to obtain high mRNA levels in cells and to deliver mRNA to a large number of cells. The intracellular uptake can be increased by electroporation, using for example a gene gun [37]. Transfer of mRNA into DCs by electroporation as a cancer vaccine has been shown to be safe in cancer patients [38,39].

mRNA formulations have been developed for in vivo delivery that increase mRNA stability and cellular uptake [40]. The most widely used of these are mRNAs formulated with lipid nanoparticles (LNP). LNPs are mixtures of cholesterol, ionizable lipids, phospholipids, PEG lipids, and a helper lipid and were initially applied for cancer immunotherapy [41,42,43]. The type of lipids and the ratio between them determines the efficiency of the formulation, and the optimal ratio for mRNA vaccines differs from that for siRNA delivery [43,44,45]. Use in the current COVID-19 pandemic has shown that LNP-formulated COVID-19 vaccines have high efficacies and are safe in humans [11,12]. In randomized controlled trials, COVID-19 mRNA vaccines reduced, compared to placebo, the proportion of participants with confirmed symptomatic COVID-19 and reduced the severity of disease. Little or no difference in serious adverse events was observed between vaccine- and placebo-treated groups [46].

### 1.3. Modulation of Innate Immune Responses to Enhance Protein Expression by mRNA Vaccines

Foreign RNA is recognized in cells by the innate immune system, which normally results in the suppression of antigen expression [47]. However, this innate immune response can also have an adjuvant effect on vaccines [48]. Several strategies have been used to optimize immunogenicity.

During in vitro transcription, the RNA-dependent RNA polymerase activity of the T7 RNA polymerase and the rebinding of the RNA allow self-priming at the 3′ end for complementary RNA synthesis resulting in double-stranded RNA (dsRNA) [49,50,51]. The dsRNA can potently activate innate immune responses [31]. During virus infections, dsRNAs can be sensed by Toll-like receptors (TLRs), retinoic acid inducible gene I (RIG-I)-like receptors, protein kinase R (PKR), oligoadenylate synthases (OAS), and NOD-, LRR-, and pyrin-domain-containing 1 (NLRP1), which results in the activation of diverse signaling cascades leading to inflammation, cell growth inhibition, or cell death [52]. dsRNA can be removed by purification of the RNA, e.g., by high-performance liquid chromatography or by cellulose-based purification [53,54]. Alternatively, dsRNA generation during in vitro transcription can be reduced by optimizing the nucleoside triphosphate ratios or constructing mutant polymerases for RNA synthesis [55,56,57]. Additionally, incorporation of modified nucleosides can reduce the immunogenicity of the single-stranded mRNA [58]. For example, pseudouridine and 1-methylpseudouridine prevent sensing by the innate immune system and enable higher antigen expression rates [59,60,61].

The DNA template might also affect innate sensing of mRNA vaccines, although most of the DNA should have been removed by purification steps or DNase treatment. Foreign cytosolic DNA is recognized by several DNA sensors like cGAS and IFI16, which mainly operate via the adaptor protein STING (for a review see [62]). However, the impact of residual DNA on mRNA vaccines has not yet been studied in detail. Residual proteins from the in vitro transcription might also stimulate innate immune responses, but such proteins are generally removed by purification. For a review of the sensing of mRNA vaccines, see [63].

### 1.4. mRNA Vaccine Storage Temperatures

The current COVID-19 mRNA vaccines require storage temperatures of –60 or –80 °C for Comirnaty or –50 °C for Spikevax [64]. These low-temperature storage requirements affect the global distribution of mRNA vaccines, particularly in low- and middle-income countries. Incorrect storage can lead to oxidation and hydrolysis of the RNA and may alter their function [65]. Lyophilization might be an alternative storage method as it may enable long-term stability at higher temperatures [66,67].

## 2. Alphavirus Replicons as Self-Amplifying Vaccine Candidates

Antigen expression correlates with the amount of mRNA delivered to antigen-presenting cells; however, mRNA is also degraded by RNases [10]. Thus, to stimulate a potent immune response, high mRNA amounts and repeated immunizations are necessary. Current COVID-19 vaccines contain 30–100 μg mRNA per dose. Self-amplifying (sa) RNA vaccines require a reduced initial amount of RNA, because the mRNA is expanded intracellularly. For example, a dose of only 10 ng saRNA was able to induce a SARS-CoV-2 specific immune response in mice [68] and 5 µg saRNA were successfully used in a clinical trial [69]. Vaccines based on this principle make use of the genome of single-stranded positive- or negative-sensed RNA viruses. Negative-sensed viruses need de novo protein synthesis mediated by their own RNA-dependent RNA polymerase to initiate transcription and require technically-demanding reverse genetics for construction. Therefore, most saRNA vaccines are based on the genome of the positive-sensed alphaviruses Venezuelan equine encephalitis virus (VEEV), Sindbis virus (SINV), or Semliki Forest virus (SFV) [70]. The genomic RNA of positive-sensed alphaviruses is translated directly.

**Figure 2:**
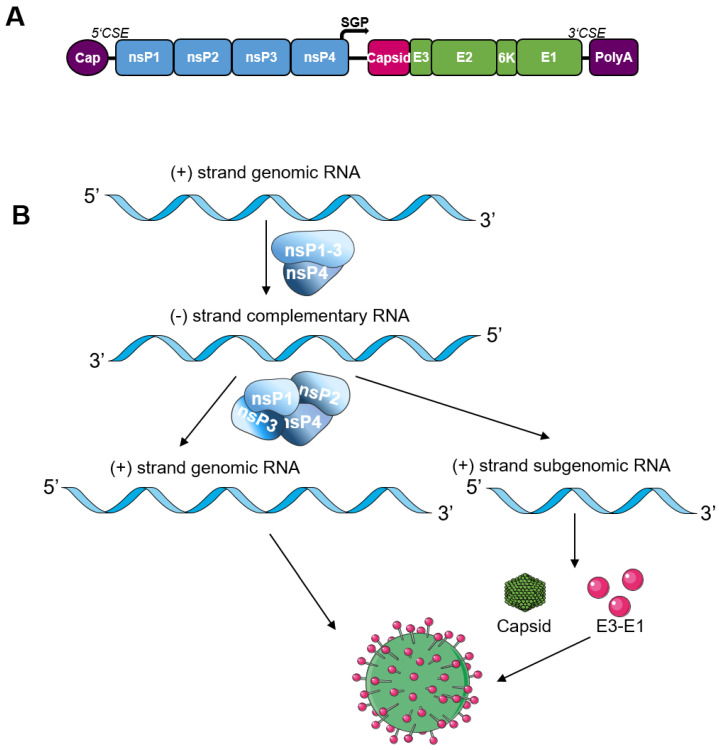
Alphavirus genome and replication: (**A**) a schematic view of an alphavirus genome. (**B**) The nsPs are directly translated from the genomic RNA and facilitate viral replication. The negative-strand intermediate serves as a template for the synthesis of genomic and subgenomic RNA. The structural proteins are translated from the latter and form an infectious virus.

Alphaviruses are enveloped, single-stranded, positive-sensed RNA viruses that belong to the *Togaviridae* family. The alphavirus genome comprises 11–12 kb with a 5′-methylguanylated cap and a 3′-poly-A tail (Figure 2A). It consists of two ORFs: the first encodes the four non-structural proteins (nsP1–nsP4) and the second encodes the five structural proteins (capsid and the envelope proteins E3-E2-6K-E1) [71].

The nsPs are directly translated as a polyprotein from the first ORF of the genomic RNA and build the replication complex (replicase), which fulfills essential functions for viral replication [72]. nsP1 is the capping enzyme and has guanine-7-methyltransferase and guanyltransferase activity [73,74,75]. nsP2 is a protease required for processing the non-structural polyprotein [76,77]. Additionally, nsP2 has RNA helicase and RNA triphosphatase activity [76,78], and it induces the shut-off of host cell protein expression [79]. nsP3 mediates virus–host and protein–protein interactions, which are essential for viral replication [80,81] and nsP4 is the viral RNA-dependent RNA polymerase [82]. The replicase complex first synthesizes a full-length negative-sensed RNA, which serves as a template for the synthesis of either the full-length genomic RNA or the subgenomic RNA (Figure 2B). In contrast to the nsPs, the structural proteins are translated as a polyprotein from the subgenomic RNA [71,83], (Figure 2B).

The genome contains several sequences that are important for RNA replication, transcription, and packaging into viral particles. These elements are called *cis*-acting or conserved sequence elements (CSEs) (Figure 2A). The 5′-UTR contains core promoter elements for both minus- and plus-strand synthesis. A 51 nt long sequence element within the nsP1 coding sequence is important for RNA amplification [84]. Similarly, 3′-CSE sequences act as a promoter in negative-strand RNA synthesis and thereby RNA amplification [85]. The presence of the 5-’ and 3′-CSEs thus ensures specific RNA amplification by the alphavirus replicase [71]. For the initiation of subgenomic RNA synthesis, the subgenomic promoter (SGP) is required as a CSE (Figure 2B). This sequence is usually located in the nsP4 coding sequence, and includes 19 nt upstream and 2–5 nt downstream of the transcription start site [86]. Additionally, packaging signals in the nsP1 or nsP2 coding sequence ensure specific packaging of the genomic RNA into virus particles [87].

## 3. Self-Amplifying RNA Vaccine Candidates

**Figure 3:**
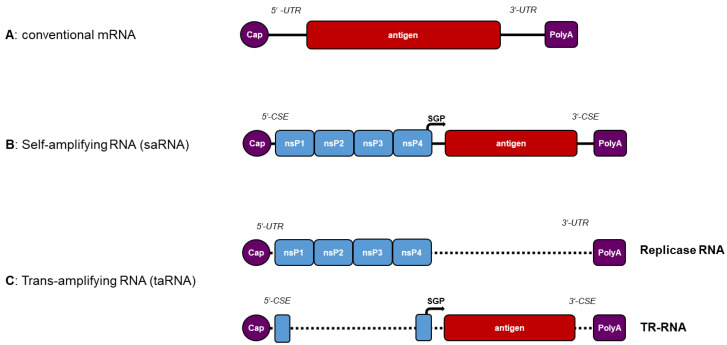
RNA vaccine approaches: (**A**) Conventional mRNAs consist of the 5′-cap, the 5′-UTR, the antigen sequence, the 3′-UTR, and the poly(A) tail. The antigen is directly translated from the mRNA in situ. (**B**) Self-amplifying RNAs additionally contain viral 5′- and 3′-CSEs and the alphavirus *nsP1–nsP4* gene. The antigen is placed under control of the subgenomic promoter (SGP). The replicase (nsP1–nsP4) is directly translated in situ and specifically amplifies the saRNA due to the CSEs. (**C**) Trans-amplifying RNA vaccines consist of two RNAs. One is a conventional mRNA encoding for the alphavirus replicase. The second is the antigen-encoding trans-replicon (TR) RNA, which is amplified by the replicase in trans.

For the construction of saRNA vaccines, the alphavirus structural proteins are replaced by the antigen gene, which is inserted under the control of the SGP (Figure 3A,B) [88]. In comparison to conventional mRNA vaccines, the addition of the alphavirus replicase gene of 7–8 kb significantly increases the length of the RNA. Moreover, the viral CSEs are used as 5′- and 3′-UTR in saRNA vaccines. Thereby, as in alphavirus replication, the replicase can amplify the saRNA and transcribe the subgenomic RNA. The antigen is then translated from the subgenomic RNA [89]. Since the replicase efficiently amplifies the antigen-encoding RNA, higher amounts of antigen will be expressed, compared to those obtained from mRNA. Thus, similar immune responses can be achieved using less RNA [90].

Several preclinical trials using saRNA vaccine candidates have been reported, predominately aiming to prevent infectious diseases. Here, the saRNA encodes the viral glycoproteins as the target for neutralizing antibodies and cellular immune responses. Alphavirus-based saRNAs were successfully tested in animal models as vaccine candidates for SARS-CoV-2 infections [68,91,92,93,94]. Recently, preclinical data for a cross-sarbecovirus saRNA vaccine candidate expressing multiple bat and human coronavirus spike antigens showed that it was able to protect against lethal heterologous infections [95]. In addition, preclinical tests have been carried out for saRNA vaccines against influenza virus [96,97,98], respiratory syncytial virus [99], rabies virus [100], Zika virus [101,102,103], Ebola virus [104], VEEV [105], and HIV-1 [106,107]. The vaccine candidates were mainly formulated with LNPs and induced high, specific antibody and T cell responses, and showed protection of mice from challenge infections. saRNA vaccines have also been adapted for use against bacterial infections [108], parasites like *Toxoplasma gondii* [104], and cancer [109]. For reviews see [42,70,110].

The preclinical development of saRNA vaccines resulted in the first clinical trials of saRNA vaccines against SAR-CoV-2 and influenza. Currently, ten clinical trials of saRNA vaccines are listed on clinicaltrials.gov, of which nine target SARS-CoV-2 (Table 1).

The application of saRNA is not limited to vaccine development; passive immunization strategies using saRNA have also been developed. A Zika virus-specific monoclonal antibody delivered by saRNA protected mice against Zika virus infection [111]. In addition, novel gene therapy approaches using mRNA or saRNA for gene replacement therapy are under development. (For a review, see [112].)

For clinical development, it remains necessary to elucidate how long RNA amplification and antigen expression continues [70]. After administration of a luciferase saRNA, expression returned to baseline levels after one month [113]. Moreover, in theory, if the saRNA expresses budding-competent viral glycoproteins, it might be released in vesicles, leading to transfer of the saRNA to additional cells [114]. This should be taken into consideration for the safety evaluation of saRNA vaccines.

## 4. Trans-Amplifying (ta) RNA Vaccine Candidates

Recently, saRNA vaccines were further developed by establishing the principle of taRNA vaccines [115]. For a taRNA vaccine, two RNAs are used. The first is an in vitro-transcribed mRNA that encodes for an alphavirus replicase and can be directly translated in situ. The second RNA, the trans-replicon (TR) RNA, encodes for the respective antigen, which is placed under control of the SGP (Figure 3C). The TR-RNA is amplified by the alphavirus replicase in trans, since it contains the alphavirus 5′- and 3′-CSEs [116].

Initially, a split replicon system called a “splitzicon” was established for VEEV. With the help of fluorescent reporter genes as antigens, the components needed for self-amplification of taRNA were identified [117]. Recently, an influenza virus taRNA vaccine candidate was constructed using a non-replicating mRNA encoding the replicase gene and a TR-RNA expressing the hemagglutinin of influenza virus. The taRNA was able to induce protective immune responses with less antigenic RNA compared to an saRNA vaccine candidate [115]. This is probably because only the short TR-RNA is amplified instead of the long saRNA. Importantly, studies have indicated that amplification of RNA by alphavirus replicases is faster and more efficient with shorter RNAs [116,118,119].

Moreover, more potent immune responses were induced by the use of a codon-optimized mRNA encoding for the alphavirus replicase [115,120]. A taRNA vaccine candidate based on another alphavirus, chikungunya virus (CHIKV), induced a potent humoral and cellular immune response and was able to protect mice from a CHIKV challenge infection [120]. Additionally, a novel bivalent taRNA vaccine candidate has been described which involves the delivery of three RNAs: one encoding the replicase and two antigen-encoding TR-RNAs [119].

In comparison to saRNA vaccines, taRNA vaccines have improved safety, manufacturability, and optimization potential [121]. The use of two RNAs minimizes the risk of transfer of the RNA to further host cells. taRNAs have a shorter RNA compared to saRNAs and, accordingly, scaled-up production is easier. However, two RNAs need to be produced and a formulation for efficient in vivo delivery has not yet been demonstrated.

## 5. saRNA Vaccine Production and Delivery

saRNAs and taRNAs are produced like mRNAs from a DNA template by in vitro transcription and the addition of a cap structure. In addition to established cap reagents, the CleanCap Reagent AU has recently been developed for the capping of alphavirus saRNAs to allow co-transcriptional capping with a natural Cap-1 structure [122].

saRNA vaccines can either be delivered as in-vitro-transcribed RNA or packaged into viral particles (VP). When packaged in VPs, they can, in principle, be considered as attenuated viruses. They should show a limited replication in humans but should still be able to induce a good immune response without signs of disease. Vaccine candidates using this delivery method have been described for CHIKV. Several deletions in CHIKV genes have been reported, such as a large deletion in nsP3 (Δ5nsP3) or in the capsid, and some of these attenuated viruses have entered clinical development [123,124,125,126]. However, VP-based delivery has several disadvantages; they frequently have suboptimal safety profiles and always retain the potential to revert to a pathogenic virus. In addition, the vector is immunogenic, which makes booster immunizations difficult. The production of saRNA vaccines by in vitro transcription, like conventional mRNA vaccines, bypasses these obstacles.

In preclinical models, immunizations with taRNA have mainly been performed by intra-dermal injection of RNA diluted in RNase-free PBS [90,120]. Similarly, naked saRNA was able to induce specific immune responses against HIV 1 and Zika virus [127,128]. However, the RNA doses were high and comparable to mRNA vaccines. Various formulations have been shown to improve in vivo delivery of mRNA. Due to the presence of the alphavirus replicase gene, saRNA vaccines are longer than mRNAs and require novel formulations for delivery [129]. Multiple approaches for saRNA formulation have been explored. In a comparison of saRNA formulations with liposomes, solid lipid nanoparticles, polymeric nanoparticles, and emulsions, the most potent induction of immune responses occurred with 1,2-dioleoyl-3-trimethylammonium-propane polymeric nanoparticles [129]. Optimized saRNA LNPs have also been developed based on LNP formulations previously optimized for siRNA and mRNA [130]. Formulation of saRNA with LNPs as a COVID-19 vaccine candidate reduced the dose required to induce a robust immune response in mice to as little as 10 ng [91]. LNP formulations could similarly be adapted for the taRNA system. Thereby, RNA stability and transmission might be improved, and robust immune responses might be stimulated with lower RNA doses and by intra-muscular application.

## 6. saRNA Vaccine Candidates and the Induction of Innate Immune Responses

As non-self molecules, RNA vaccines are sensed by cellular pathways and are effective activators of the innate immune system [130]. As depicted above for mRNA vaccines, several approaches to circumvent innate immune activation can be applied; however, for sa/taRNA vaccines, nucleoside modifications will be lost during the amplification step and will be of less benefit [108,111]. In a taRNA vaccine, the replicase mRNA could be nucleoside-modified; however, the effects remain to be evaluated. In addition, the RNAs can be initially purified to reduce innate sensing [122].

In contrast to mRNA vaccines, the intracellular RNA amplification results in dsRNA and thus a stronger activation of innate immune responses. RNA can be recognized by multiple pattern-recognition receptors including TLR3, TLR7, RIG-I-like receptors, melanoma differentiation-associated protein 5 (MDA5), PKR, and OAS [131]. The resulting signaling cascades lead to the production of type I interferons (IFN) and pro-inflammatory cytokines [24]. Although the innate response has an adjuvant effect which can promote the specific immune response, it can also induce RNA degradation and thereby reduce antigen expression [132].

Strategies to reduce the IFN activation have been described for saRNA vaccines. For example, the expression of proteins derived from viruses that avoid immune sensing can inhibit innate responses [133]. Encoding the E3, K3, and B18R proteins of vaccinia virus and the non-structural protein 1 of influenza A virus on a separate mRNA increased antigen expression in vitro and in vivo [134,135]. Similarly, innate response-inhibiting proteins encoded in cis on an saRNA were able to increase the stimulated immune responses [136].

Alphaviruses counteract innate immune responses by shutting off host cell transcription and translation [79]. Thereby, IFN production is also reduced. Additionally, alphavirus infection and replicase expression are cytotoxic and induce host cell apoptosis [137]. The replicase nsPs contain elements that influence RNA amplification and the host cell response. nsP2 induces host cell shut-off leading to cell death [79]. The precise effects of this on vaccine applications remain to be elucidated. Interestingly, mutations in the nsP2 of CHIKV have been described that reduce the cytopathic effect [138]. Moreover, in an in vitro approach, mutations in the VEEV nsPs have been identified that enhance the synthesis of subgenomic RNA in situ [109]. During adaption of CHIKV to A549 cells, two mutations in the replicase gene occurred that increased viral replication [139]. Accordingly, the replicase of the sa/taRNA vaccine candidates could be further optimized for higher antigen expression and immunity.

Besides strategies to reduce IFN activation, the modulation of immune responses after saRNA vaccination through the use of adjuvants has also been explored. For example, saRNA formulated with a cationic nanoemulsion based on the adjuvant MF59 or formulations with TLR agonists have been evaluated [136,140]. Although potent immune responses were induced, direct benefits remain to be demonstrated and clinical data is lacking. Moreover, LNP formulation has adjuvant effects. A comparison of intra-dermal electroporation with LNP delivery indicated that the strong induction of innate immune responses by LNPs reduced antigen expression from saRNA in mouse skin [141].

## 7. saRNA Vaccine Application for Human Use

The preclinical development of saRNA vaccines resulted in the first clinical trials of saRNA vaccines against SAR-CoV-2 and influenza. The clinical trial data for completed saRNA-based COVID-19 vaccine candidates look encouraging, and a first phase I trial was conducted recently [69]. The VEEV-based saRNA vaccine (LNP-nCoVsaRNA) expressing the pre-fusion stabilized spike glycoprotein of SARS-CoV-2 was well tolerated with no serious adverse events related to vaccination. Although seroconversion rates did not reach 100%, specific antibody concentrations among the seropositive participants were similar to values derived from convalescent sera. However, human responses to SARS-CoV-2 were significantly lower than those predicted by small animal models [69]. Another VEEV-based saRNA vaccine candidate (ARTC-021) expressing the SARS-CoV-2 spike glycoprotein was also found to be safe and had a 100% seroconversion rate. Anti-spike IgG titers were equal to those in COVID-19 convalescent plasma [142]. A larger clinical trial is currently ongoing with ARTC-021 as a booster vaccine (NCT05012943). These examples show that saRNA technology is entering clinical development, but further improvements are necessary to generate potent vaccines.

## 8. Conclusions

The use of mRNA vaccines during the COVID-19 pandemic has demonstrated their feasibility in preventing infectious diseases. saRNA vaccine candidates will further improve this strategy and they hold the promise of being effective with less RNA. However, further research is still required, in particular, to improve the efficiency of RNA delivery and investigate the role of innate sensing. It is also still not certain whether using less RNA, which is amplified in situ, is clinically advantageous compared to standard mRNA vaccines. Comparative preclinical studies demonstrating the advantages of saRNA over mRNA were performed in mouse models, which do not properly recapitulate the innate immune responses in humans. The first clinical trials of saRNA vaccines have just been completed and direct comparisons of mRNA with saRNA remain to be carried out. Like RNA vaccines, saRNA vaccines might not be limited to infectious diseases but might also be used in gene therapy, to fight cancer, or to deliver protein-based therapeutics.

## Figures and Tables

**Table 1:** Status of saRNA vaccine clinical trials against infectious diseases.

Vaccine	Target	Status	Sponsor	clinicaltrials.gov Identifier
ARCT-154-01	SARS-CoV-2	Phase 1/2/3 Active, not recruiting	Vinbiocare Biotechnology Joint Stock Company	NCT05012943
ARCT-165, ARCT-154, ARCT-021	SARS-CoV-2	Phase 1/2 Recruiting	Arcturus Therapeutics, Inc.	NCT05037097
saRNA-LNP based on VEEV	SARS-CoV-2	Phase 1 Active, not recruiting	National Institute of Allergy and Infectious Diseases (NIAID)	NCT04776317
LNP-nCOV saRNA-02 Vaccine	SARS-CoV-2	Phase 1 Recruiting	MRC/UVRI and LSHTM Uganda Research Unit	NCT04934111
GRT-R912, GRT-R914, and GRT-R918	SARS-CoV-2	Phase 1 Recruiting	Gritstone bio, Inc.	NCT05435027
GRT-R910	SARS-CoV-2	Phase 1 Active, not recruiting	Gritstone bio, Inc.	NCT05148962
CoV2 SAM (LNP)	SARS-CoV-2	Phase 1 Completed	GlaxoSmithKline	NCT04758962
AAHI-SC2, AAHI-SC3	SARS-CoV-2	Phase 1/2 Recruiting	ImmunityBio, Inc.	NCT05370040
ARCT-021	SARS-CoV-2	Phase 2 Terminated	Arcturus Therapeutics, Inc.	NCT04668339
PF-07852352, PF-07836391, PF-07836394, PF-07836395, PF-07836396, PF-07867246	Influenza	Phase 1 Recruiting	Pfizer	NCT05227001

## Data Availability

Not applicable.
